# Benefits of the Hydrophobic Surface for CH_3_NH_3_PbI_3_ Crystalline Growth towards Highly Efficient Inverted Perovskite Solar Cells

**DOI:** 10.3390/molecules24102027

**Published:** 2019-05-27

**Authors:** Yang Li, Zheng Xu, Suling Zhao, Dandan Song, Bo Qiao, Youqin Zhu, Juan Meng

**Affiliations:** 1 Key Laboratory of Luminescence and Optical Information, Beijing Jiaotong University, Ministry of Education, Beijing 100044, China; 15118429@bjtu.edu.cn (Y.L.); slzhao@bjtu.edu.cn (S.Z.); ddsong@bjtu.edu.cn (D.S.); boqiao@bjtu.edu.cn (B.Q.); 15118427@bjtu.edu.cn (Y.Z.); 17118459@bjtu.edu.cn (J.M.); 2 Institute of Optoelectronics Technology, Beijing Jiaotong University, Beijing 100044, China

**Keywords:** inverted perovskite solar cells (PSCs), hydrophobic surface, small molecule, hole-transporting material (HTM), crystalline growth

## Abstract

In inverted perovskite solar cells (PSCs), high-quality perovskite film grown on hole-transporting material (HTM) with pinhole-free coverage and a large grain size is crucial for high efficiency. Here, we report on the growth of pinhole-free and large grain CH_3_NH_3_PbI_3_ crystals favored by a hydrophobic small molecular HTM, namely, 4,4′-Bis(4-(di-p-toyl)aminostyryl)biphenyl (TPASBP). The hydrophobic surface induced by TPASBP suppressed the density of the perovskite nuclei and heterogeneous nucleation, thus promoting the perovskite to grow into a dense and homogeneous film with a large grain size. The CH_3_NH_3_PbI_3_ deposited on the TPASBP exhibited better crystallization and a lower trap density than that on the hydrophilic surface of indium tin oxide (ITO), resulting in a significant reduction in carrier recombination. Combined with the efficient hole extraction ability of TPASBP, a high efficiency of 18.72% in the inverted PSCs fabricated on TPASBP was achieved.

## 1. Introduction

Between 2009 and 2019, the efficiency of perovskite solar cells (PSCs) was boosted from 3.8% to over 24% [1,2,3,4,5] in individual devices. In a typical PSC, whether a regular or inverted structure, a several hundred nanometer thick perovskite layer is sandwiched between the electron and the hole transporting layers. For the so-called inverted structure, a multilayer stack of transparent electrode/hole-transporting material (HTM)/perovskite/electron-transporting material (ETM)/metal electrode is fabricated, where the HTM, an important component of PSCs, plays a significant role in hole extraction and transport [6,7], along with affecting the growth of perovskite films [8,9,10].

Currently, the HTMs employed in inverted PSCs are mainly p-type wide band-gap semiconductors, which can be divided into conductive polymers and inorganic p-type semiconductors. In conductive polymers, poly(3,4-ethylenedioxythiophene):poly(styrenesulfonic acid) (PEDOT:PSS) is a widely used HTM due to its favorable conductivity and high transmittance [11,12]. With the incessant optimization of high efficiency PSCs, the power conversion efficiency (PCE) of inverted PSCs based on PEDOT:PSS has increased from 3.9%, which was reported by Chen et al. for the first time in 2013 [13], to 18.1% achieved by Im et al. in 2015 [14]. Besides PEDOT:PSS, poly(p-phenylene) (PPP), poly(*N*,*N*′-bis(4-butylphenyl)-*N*,*N*′-bis(phenyl)benzidine) (poly-TPD), and poly(bis(4-phenyl)(2,4,6-trimethylphenyl)amine) (PTAA) are also excellent HTMs in inverted PSCs, with PCEs of 16.7% [10], 19.1% [15], and 21.51% [16], respectively. As for inorganic HTMs, both N_i_O_x_ (widely studied) and C_u_X (X = SCN, I, O_x_) are good HTM candidates as well. Han et al. reported a high PCE of 18.3% by implementing the co-p^+^-doping strategy for N_i_O_x_ by adding Mg^2+^ and Li^+^ [17]. Bian et al. obtained the PSCs with a structure of indium tin oxide (ITO)/C_u_O_x_/CH_3_NH_3_PbI_3-x_Cl_x_/[6,6]-phenyl-C61-butyric acid methyl ester (PCBM)/C_60_/BCP/Ag, yielding a PCE as high as 19.0% [18].

There is no doubt that efficient HTMs with a good ability to carry out hole extraction and transport are important for PSCs [19,20,21]. At the same time, it is suggested that the interaction between the bottom charge-transporting layer and the perovskite also plays a role in determining the photovoltaic performance of PSCs [22]. Huang et al. reported the growth of perovskite grains with a high average aspect ratio on a range of non-wetting HTMs, yielding a best PCE of 18.1% [8]. Bo et al. used a thermos-cleavable fullerene derivative to modify the TiO_2_ surface, which showed exceptional resistance against polar solvents, and thus, the resulting perovskite film was of high quality with less pinholes [23]. Other materials, such as Trux-OMeTAD [20], perylene [24], and ITIC [25], have also been applied as HTM or interfacial modifications in PSCs to promote the growth of perovskite on them.

Here, we explored the functions of a small molecular HTM, 4,4′-Bis(4-(di-p-toyl)aminostyryl)biphenyl (TPASBP) (shown in Figure S1a), that affect perovskite growth and device performance. It was found that the hydrophobic surface of TPASBP is crucial for homogeneous, pinhole-free CH_3_NH_3_PbI_3_ film with larger crystallites, and it induced a lower hole trap density compared to CH_3_NH_3_PbI_3_ film on ITO. Thus, the recombination of PSCs fabricated on TPASBP declined dramatically. Along with a more effective hole extraction ability, PSCs fabricated with the inverted structure using TPASBP as the HTM showed the best PCE of 18.72% along with an average PCE of 17.23%.

## 2. Materials and Methods

### 2.1. Materials

PbI_2_ and CH_3_NH_3_I were purchased from Xi’an Polymer Light Technology Corp. PCBM was purchased from Nano-C. TPASBP was synthesized according to the route reported previously [26]. All the materials were used as received.

### 2.2. Device Fabrication

ITO glasses were cleaned in ultrasonic baths containing glass lotion, de-ionized water, and ethanol for 20 min each, and then blow-dried by nitrogen gas. All pre-cleaned ITO substrates were treated by UV-ozone for 8 min before device fabrication and then transferred into a glove box filled with nitrogen. For the PSCs with TPASBP as the HTM, TPASBP was dissolved in a mixture of chlorobenzene and tetrahydrofuran (1:1, *v*/*v*) with a concentration of 5 mg/mL. The as-prepared TPASBP solution was firstly spun onto the ITO with the spin speed of 3000 revolutions per minute (rpm) for 40 s and then annealed at 100 °C for 10 min, which afforded a thickness of ∼20 nm. Different thicknesses of TPASBP can be obtained by varying the spin speed. The precursor solution of CH_3_NH_3_PbI_3_ was composed of PbI_2_ and CH_3_NH_3_I (1:1 molar ratio) with a concentration of 43 w%, i.e., 645.4 mg PbI_2_ and 222.6 mg CH_3_NH_3_I were dissolved in a mixture of 700 μL γ-butyrolactone (GBL) and 300 μL dimethyl sulfoxide (DMSO). Then, the perovskite film was deposited by the one-step spin-coating method reported previously [27,28] but using chlorobenzene as an anti-solvent. The perovskite-precursor coated substrates were next annealed at 100 °C for 5 min. A 30 mg/mL PCBM chlorobenzene solution was then spin-coated onto the CH_3_NH_3_PbI_3_ layer at 2000 rpm for 60 s. Finally, an Al electrode (100 nm) was deposited by thermal evaporation under a 4 × 10^−4^ Pa vacuum condition. The area of the solar cell was 4 mm^2^.

### 2.3. Measurements and Characterization

The current density-voltage (J-V) characteristics were recorded using a Keithley 4200 (Tektronix Inc., Beaverton, OR, USA) source measurement unit under 100 mW/cm^−2^ illumination (AM 1.5 G) generated by an ABET Sun 2000 solar simulator (Abet Technologies, Inc., Milford, CT, USA) in air. The dark current-voltage (I-V) for hole-only devices, dark (J-V) for PSCs, and capacitance-voltage (C-V) characteristics were demonstrated by the Keithley 4200 source measurement unit under dark conditions. All the measurements above were done outside the glove box and without any encapsulation of the devices, under constant exposure to ambient atmosphere.

Contact angles were measured using an OCA20 instrument (DataPhysics Instruments GmbH, Filderstadt, Germany), and the system was maintained at an ambient temperature and saturated humidity. Scanning electron microscopy (SEM) was performed using a Hitachi, S-4800 SEM microscope (Hitachi, Ltd., Tokyo, Japan). Atomic force microscopy (AFM) was performed using a multimode Nanoscope IIIa microscope (Veeco Corp., Plainview, NY, USA), operated in tapping mode with a scan size of 2 × 2 μm^2^. The absorption and transmittance spectra were measured by a Shimadzu UV-3101 PC spectrometer (Shimadzu Corp., Kyoto, Japan). X-ray diffraction (XRD) and two-dimensional grazing-incident wide-angle X-ray scattering (GIWAXS) were measured using 1W1A at the Beijing Synchrotron Radiation Facility (BSRF) (The Institute of High Energy Physics of the Chinese Academy of Sciences, Beijing, China). The steady-state photoluminescence (PL) was obtained with a Horiba Fluorolog-3 spectrofluorometer (Horiba, Ltd., Kyoto, Japan)—the excitation wavelength was 505 nm. Time-solved photoluminescence (TRPL) was recorded at the emission wavelength of 760 nm and the samples were excited by a 485 nm diode laser.

## 3. Results and Discussion

### 3.1. Characterization of the As-Prepared Perovskite Films

**Figure 1:**
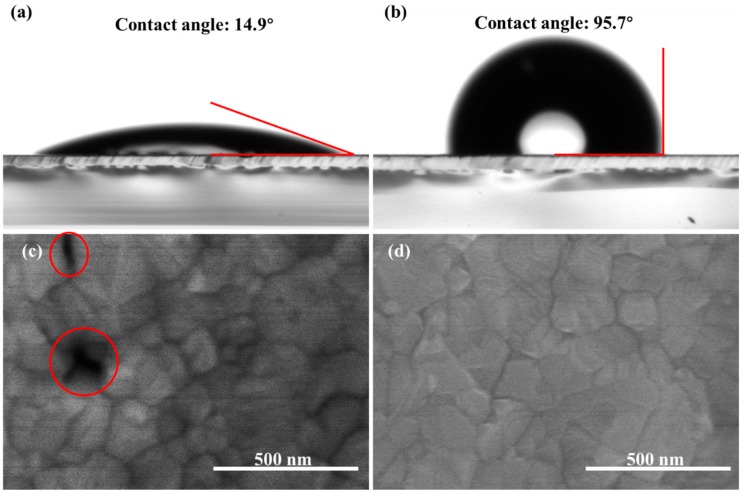
Contact angles of (**a**) indium tin oxide (ITO) and (**b**) ITO/4,4′-Bis(4-(di-p-toyl)aminostyryl)biphenyl (TPASBP) surfaces, and corresponding top-view SEM images of the perovskite films grown on (**c**) bare and (**d**) TPASBP-covered ITO substrates; the red circles in Figure 1c indicate pinholes in perovskite film.

The hydrophobic property of the substrates is crucial for the nucleation of the perovskite [8,20]. Hence, the water contact angle images on the ITO and TPASBP surfaces were characterized, as shown in Figure 1a,b. The contact angles of water on the two different surfaces were 14.9° and 95.7° for the ITO and TPASBP, respectively. The larger contact angle means that the TPASBP is much more hydrophobic than a bare ITO surface. Because of the hydrophobic property of TPASBP, it hardly dissolves in many polar solvents like GBL, DMSO, *N*,*N*-dimethylformamide (DMF), etc. (as shown in Figure S1c). Hence, the hole-transporting layer and the CH_3_NH_3_PbI_3_ layer can be fabricated consecutively on ITO by a solution process. Moreover, the top-view SEM images of the CH_3_NH_3_PbI_3_ films on bare ITO and TPASBP (shown in Figure 1c,d) revealed a clear correlation between the hydrophobic property of the substrate surface and the CH_3_NH_3_PbI_3_ film morphology. It is obvious that there are clearly discerned pinholes in the perovskite film on bare ITO, whereas dense perovskite film without pinholes was obtained on TPASBP. Meanwhile, the average size of the CH_3_NH_3_PbI_3_ grains on TPASBP was ∼203 nm, which was larger than that on ITO (average grain size ∼160 nm). Figure S2 shows the corresponding grain size distribution of Figure 1c,b. The morphologies of CH_3_NH_3_PbI_3_ films on different substrates were further investigated by using AFM (as shown in Figure S3), where the CH_3_NH_3_PbI_3_ film based on hydrophobic TPASBP showed a smoother surface (root mean square (RMS) = 11.02 nm) relative to that on ITO (RMS = 14.69 nm). As shown in Figure S4, compared to hydrophilic ITO, the hydrophobic surface of TPASBP induced a lower density of perovskite nuclei after spin-coating the perovskite precursor, thus promoting the perovskite to grow into compact films with large grains after thermal annealing. Meanwhile, due to the reduction of perovskite nuclei on the substrate, the heterogeneous nucleation of perovskite was suppressed, and a much smoother perovskite film was realized.

**Figure 2:**
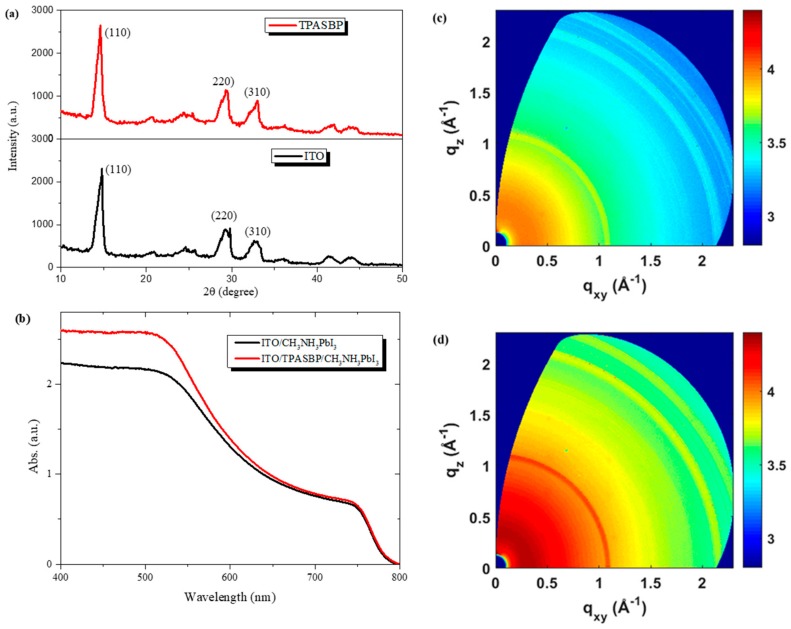
(**a**) X-ray diffraction (XRD) patterns, and (**b**) UV–vis absorption spectra of CH_3_NH_3_PbI_3_ films on ITO and TPASBP substrates, (**c**) and (**d**) two-dimensional grazing-incident wide-angle X-ray scattering (GIWAXS) patterns of CH_3_NH_3_PbI_3_ films on ITO and TPASBP, respectively.

To gain further insight into the effect of hydrophobic TPASBP surfaces on CH_3_NH_3_PbI_3_ crystallization, the XRD patterns of CH_3_NH_3_PbI_3_ films on ITO and TPASBP are shown in Figure 2a. No significant change in the diffraction peak ratio was observed, indicating the same crystal orientation of CH_3_NH_3_PbI_3_ film on both ITO and TPASBP. However, the X-ray diffraction peaks of CH_3_NH_3_PbI_3_ (110), (220), and (310), were more intense and sharper for perovskite film on hydrophobic TPASBP. In addition, two-dimensional GIWAXS patterns of perovskite films on ITO and TPASBP were measured and are shown in Figure 2c,d respectively. For the perovskite film deposited on TPASBP, the feature of CH_3_NH_3_PbI_3_ crystalline domains was clearer and the two-dimensional GIWAXS pattern exhibited stronger intensity at the diffraction peaks, indicating that the perovskite film on TPASBP showed finer ordering structures and better crystallization than that on ITO. The results from XRD and GIWAXS imply that the crystallization of CH_3_NH_3_PbI_3_ grown on the hydrophobic TPASBP was greatly improved in comparison with that on bare ITO. Hence, on the basis of improved CH_3_NH_3_PbI_3_ morphology and crystallization, the light absorption of perovskite film on TPASBP is enhanced in the whole wavelength range from 400 to 800 nm, especially from 400 to 550 nm, as presented by the UV–vis absorption spectra of perovskite films on ITO and TPASBP in Figure 2b.

**Figure 3:**
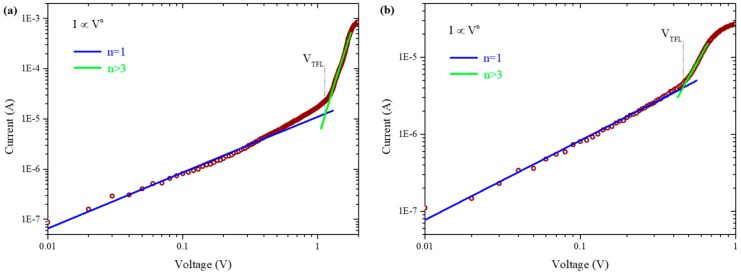
Characteristic current-voltage (I-V) trace (red markers) showing two different regimes for (**a**) ITO/CH_3_NH_3_PbI_3_/Au, and (**b**) ITO/TPASBP/CH_3_NH_3_PbI_3_/Au. A linear ohmic regime (I ∞ V, blue line) is followed by the trap-filled regime, marked by a steep increase in current (I ∞ V^n^ ^> 3^, green line).

Large grain size and compact CH_3_NH_3_PbI_3_ film tends to yield less trap states, which are beneficial for high-performance PSCs [8,29]. Hence, hole trap density in the CH_3_NH_3_PbI_3_ films was quantified by the dark I-V analysis for hole-only devices. Figure 3a,b show the I-V curves of the hole-only devices based on ITO and ITO/TPASBP substrates in the dark. It is clear that the linear regime (blue line) at low voltage indicates an ohmic response of the hole-only devices. With the increase of voltage, the current exhibits a rapid nonlinear rise (green line), presenting the transition to the trap-filled limit (TFL) regime in which all the available trap states are filled by the injected carriers. The TFL voltage V_TFL_ is determined by the trap density [30,31]:

(1) $$V_{\mathrm{TFL}}=\frac{{\mathrm{en}}_{\mathrm{trap}}L^{2}}{2{\varepsilon \varepsilon }_{0}}$$

where e is the elementary charge of the electron, L is the thickness of the CH_3_NH_3_PbI_3_ film, ε is the relative dielectric constant of CH_3_NH_3_PbI_3_ (ε = 30) [32], ε_0_ is the vacuum permittivity, and n_trap_ is the trap density. The onset voltage V_TFL_ is linearly proportional to the density of trap states, n_trap_; hence, the trap density can be calculated using Equation (1). Based on Figure 3a,b the V_TFL_ values for the CH_3_NH_3_PbI_3_ films grown on ITO and TPASBP are 1.12 and 0.46 V, respectively. Correspondingly, the CH_3_NH_3_PbI_3_ film on bare ITO yields a hole trap density of 1.48 × 10^16^ cm^−3^, whereas the CH_3_NH_3_PbI_3_ film deposited on TPASBP has a hole trap density as low as 6.10 × 10^15^ cm^−3^.

On the basis of the above results, it was clear that a hydrophobic surface provided by TPASBP was beneficial for the formation of dense pinhole-free CH_3_NH_3_PbI_3_ film with a larger grain size relative to hydrophilic substrates, thus resulting in better crystallization of CH_3_NH_3_PbI_3_. Meanwhile, due to the improvement of CH_3_NH_3_PbI_3_ morphology and crystallization, the trap density was reduced significantly for the perovskite film fabricated on TPASBP compared with that on ITO, which induced less recombination loss in the PSCs.

**Figure 4:**
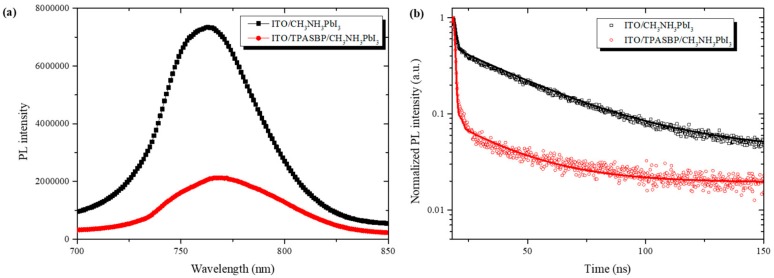
(**a**) Steady-state PL spectra, and (**b**) time-resolved PL (TRPL) decay curves of CH_3_NH_3_PbI_3_ films on ITO and ITO/TPASBP substrates.

Besides the hydrophobicity, TPASBP also has high hole mobility because of its linear π-conjugated structure, which has been illustrated before elsewhere [26,33]. Meanwhile, the results of steady-state PL and time-resolved PL (TRPL) shown in Figure 4 also revealed that the hole-extraction efficiency at the ITO/CH_3_NH_3_PbI_3_ interface was improved by using TPASBP. As presented in Figure 4a, a much stronger quenching effect on the PL of the CH_3_NH_3_PbI_3_ film on TPASBP was observed in comparison with that on ITO, suggesting an enhanced hole-extraction ability of TPASBP as an HTM. In order to further evaluate the hole-extraction rate, TRPL was performed and the decay curves are shown in Figure 4b. By a biexponential fitting of the dynamic TRPL data, the CH_3_NH_3_PbI_3_ film on ITO exhibited a longer lifetime of 32.26 ns, whereas the lifetime of CH_3_NH_3_PbI_3_ film grown on TPASBP was 21.87 ns. More details about carrier lifetime can be found in Table S1. The smaller PL lifetime of CN_3_NH_3_PbI_3_ fabricated on TPASBP indicates the reduced carrier recombination due to the more efficient hole extraction and transport in CH_3_NH_3_PbI_3_ film. The high mobility and the enhanced hole-extraction ability of TPASBP contributes to the high carrier collection efficiency of PSCs, thus leading to an enhancement of short-circuit current (Jsc).

### 3.2. Photovoltaic Performance of PSCs

**Figure 5:**
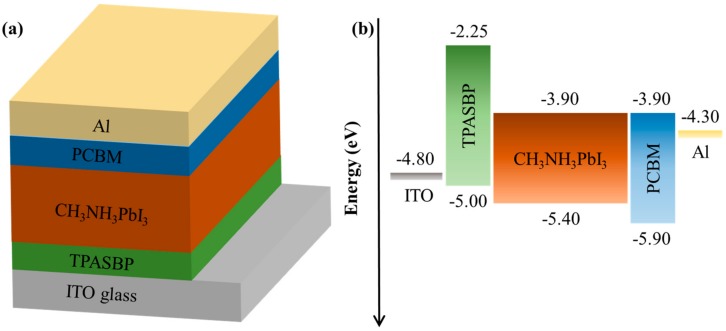
(**a**) Schematic device structure of the inverted PSCs constructed by ITO/TPASBP/CH_3_NH_3_PbI_3_/PCBM/Al, (**b**) schematic energy level diagram of each layer.

The influence of TPASBP on device performance was evaluated in inverted PSCs with a structure shown in Figure 5a, in which all layers between the electrodes were fabricated by spin-coating. The relative energy levels are presented in Figure 5b, where it can be seen that the highest occupied molecular orbital (HOMO) of TPASBP is between the work function of ITO and the valence band (VB) of CH_3_NH_3_PbI_3_, implying that the potential energy loss at the interface of TPASBP/CH_3_NH_3_PbI_3_ can be lowered.

**Figure 6:**
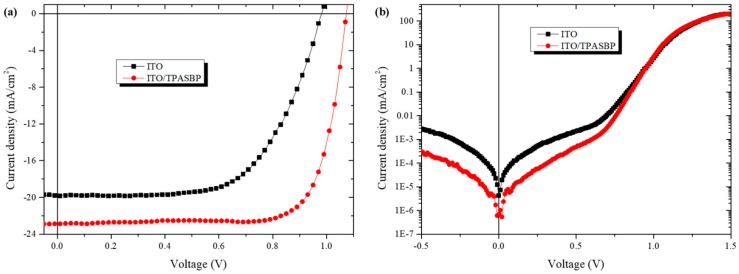
(**a**) Current density-voltage (J-V) characteristics of the best PSCs based on bare ITO and TPASBP-covered ITO under irradiation of 100 mW/cm^−2^, (**b**) dark J-V characteristics of the PSCs with and without TPASBP.

The J-V characteristics of the inverted PSCs fabricated on TPASBP under 100 mW/cm^−2^ illumination (AM 1.5 G) are shown in Figure 6a. The same irradiation was carried out on PSCs based on bare ITO for comparison. PSCs fabricated on TPASBP afforded the best PCE of 18.72% with a Jsc of 22.87 mA/cm^−2^, an open circuit voltage (Voc) of 1.07 V, and a fill factor (FF) of 76.47% (listed in Table 1). The histogram of PCE shown in Figure S5 also indicates that inverted PSCs based on TPASBP exhibit good reproducibility, with an average efficiency of 17.23%. Meanwhile, the dependence of PSCs performance on the thickness of TPASBP is plotted in Figure S6, which reveals a thickness of 20 nm is optimal for TPASBP.

Dark J-V characteristics shown in Figure 6b indicates that the leakage current density at the low voltage scale was weakened dramatically in the PSCs with perovskite film grown on TPASBP. It has been reported that the leakage current is determined by R_Sh_ and consequent charge carrier recombination [34]. A reduction of leakage current means an increase of R_Sh_ and a reduction of charge carrier recombination in the device. The result of dark J-V was in good agreement with that of the reverse saturation current, which is discussed in the following part, revealing the remarkable role of TPASBP in preventing recombination in PSCs.

The improved FF and Voc strongly imply a reduction in recombination loss as a result of the utilization of TPASBP in inverted PSCs. The planar structured PSCs can be treated as single heterojunction diode. The electric parameters of the PSCs, including series resistance (Rs), and reverse saturation current density (J_0_), can be extracted from the J-V curves shown in Figure 6a according to the diode equation [35,36]:

(2) $$J=J_{L}-J_{0}\left[\exp\left(\frac{e\left(V+J\times R_{S}\right)}{{\mathrm{AK}}_{B}T}\right)-1\right]-\frac{V+J\times R_{S}}{R_{\mathrm{sh}}}$$

where A is the ideality factor of the heterojunction, and K_B_ and T are the Boltzmann constant and absolute temperature, respectively. Equation (2) can also be written as (where R_Sh_ is large enough):

(3) $$-\frac{\mathrm{dV}}{\mathrm{dJ}}=\frac{{\mathrm{AK}}_{B}T}{e}{\left(J_{\mathrm{SC}}-J\right)}^{-1}+R_{S}$$

(4) $$\ln\left(J_{\mathrm{SC}}-J\right)=\frac{e}{{\mathrm{AK}}_{B}T}\left(V+J\times R_{S}\right)+{\mathrm{lnJ}}_{0}.$$

The calculated values of A, Rs, J_0_ are listed in Table 2. For a well-behaved heterojunction solar cell, the ideality factor is typically in the range of 1.3 ˂ A ˂ 2 [36]. In the PSCs with perovskite film grown on TPASBP, the ideality factors obtained from Equations (3) and (4) are 1.56 and 1.72, respectively, which indicate that the PSCs based on TPASBP work well on the basis of a heterojunction model. It is also clear that Rs is much lower in the PSCs fabricated on TPASBP (1.19 Ω cm^2^) than the PSCs fabricated on ITO (5.68 Ω cm^2^). As also shown in Table 2, J_0_ for the PSCs with perovskite film grown on TPASBP is ∼2 orders of magnitude lower than those PSCs on ITO. J_0_ correlates with the carrier recombination in heterojunction solar cells, where a lower J_0_ corresponds to lower carrier recombination in PSCs. Therefore, the recombination in inverted PSCs is greatly lowered by using TPASBP. Meanwhile, according to the equation Voc = (AK_B_T/e)ln(Jsc/J_0_+1), smaller J_0_ also leads to larger Voc; hence, Voc in the PSCs with perovskite film deposited on TPASBP is higher than that on ITO.

**Figure 7:**
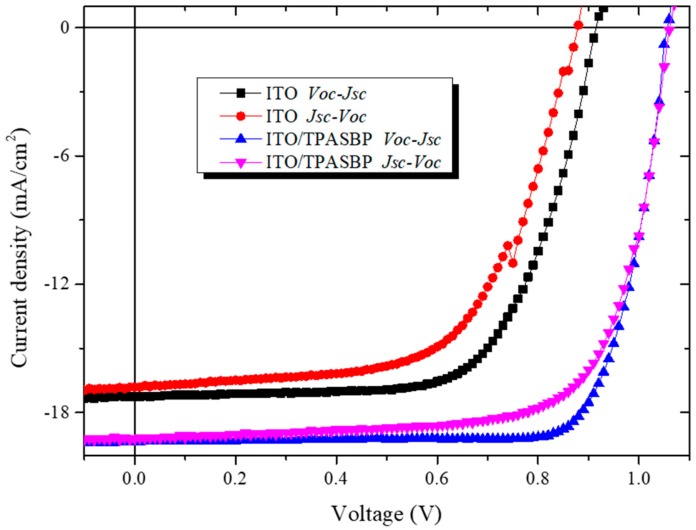
J-V characteristics of inverted PSCs with different scanning directions.

Finally, the hysteresis of these devices was investigated, as displayed in Figure 7. According to the device performance of PSCs with respect to the scan direction under a 10 mv per point (voltage scan step) condition, it was clear that the PSCs based on ITO had much more serious J-V hysteresis while the PSCs fabricated on TPASBP gave almost the same values of Voc and Jsc with a small difference of FF under different scan modes. The restrain of hysteresis by TPASBP can be attributed to the improved quality of the CH_3_NH_3_PbI_3_ film and the significant decrease of recombination in the devices, which derived from the pinhole-free morphology and lower trap density of CH_3_NH_3_PbI_3_ grown on hydrophobic TPASBP, as described above.

## 4. Conclusions

In conclusion, the functions of a hydrophobic small molecular hole-transporting material in perovskite growth and device performance were explored in inverted PSCs. The hydrophobic TPASBP promoted the growth of CH_3_NH_3_PbI_3_ film deposited on it with pinhole-free coverage and a large grain size, leading to a decreased trap density and thus a remarkable reduction of recombination loss. TPASBP as an HTM also enabled effective hole extraction. Correspondingly, the PSCs with hydrophobic small molecular material as an HTM achieved a PCE as high as 18.72%. This work highlights the benefits of a hydrophobic surface for the crystalline growth of perovskite film, which provides an efficient way to obtain high-quality perovskite film for high performance inverted PSCs.

## Figures and Tables

**Table 1:** The photovoltaic parameters of PSCs with perovskite film fabricated on ITO and ITO/TPASBP, respectively.

PSCs	V_oc_ [V]	J_sc_ [mA/cm^2^]	FF [%]	PCE [%]	Average PCE ^a^ [%]
ITO	0.98	19.72	62.35	12.05	11.07± 0.97
ITO/TPASBP	1.07	22.88	76.47	18.72	17.23 ± 0.79

^a^ the average PCE of PSCs based on ITO was obtained from 8 identical cells and the average PCE of PSCs based on TPASBP was obtained from 13 identical cells.

**Table 2:** Fitted electrical parameters of PSCs with perovskite film fabricated on ITO and ITO/TPASBP, respectively.

PSCs	A_1_^a^	A_2_^b^	R_S_ (Ωcm^2^)	J_0_ (mAcm^−2^)
ITO	2.27	2.22	5.68	2.27 × 10^-4^
ITO/TPASBP	1.56	1.72	1.19	2.38 × 10^-6^

**^a^** A_1_ and (series resistance) R_s_ were obtained from Equation (3), **^b^** A_2_ and (reverse saturation current density) J_0_ were obtained from Equation (4).

## Supplementary Materials

The following are available online at https://www.mdpi.com/1420-3049/24/10/2027/s1, Figure S1: (a) Chemical structure of TPASBP, (b) transmittance spectra of TPASBP/ITO grass with ITO/grass as reference and UV-vis absorption spectra of TPASBP on ITO, (c) TPASBP solutions in DMF, GBL, and DMSO, respectively, with a concentration of 5 mg/mL, Figure S2: (a) Grain size distribution of the perovskite film in Figure 1(c), (b) grain size distribution of the perovskite film in Figure 1(d), Figure S3: 3D-AFM topography images of the perovskite films deposited on (a) bare and (b) TPASBP-covered ITO substrates, Figure S4: Depiction of the growth process for perovskite films on (a) hydrophilic ITO and (b) hydrophobic TPASBP, Figure S5: Distributions of (a) Jsc, (b) Voc, (c) FF, and (d) PCE obtained from eight identical cells for PSCs based on ITO and 13 identical cells for PSCs based on TPASBP, Figure S6: Dependence of the main parameters of the device performance on the thickness of TPASBP layer, Table S1: Time-resolved PL measurements, Table S2: The photovoltaic parameters of PSCs with different thicknesses of TPASBP.
