# Supplementary material for: Benefits of the Hydrophobic Surface for CH3NH3PbI3 Crystalline Growth towards Highly Efficient Inverted Perovskite Solar Cells
Source: Molecules. 2019 May 27;24(10):2027. doi: 10.3390/molecules24102027 (PMC6571817; doi:10.3390/molecules24102027)
Supplement: Supplementary file 1 [file molecules-24-02027-s001.pdf]

Supplementary Materials

Benefits of the hydrophobic surface for $CH_3NH_3PbI_3$ crystalline growth towards highly efficient inverted perovskite solar cells

Yang Li [1,2], Zheng Xu [1,2,*], Suling Zhao [1,2], Dandan Song [1,2], Bo Qiao [1,2], Youqin Zhu [1,2], and Juan Meng [1,2]

[1] Key Laboratory of Luminescence and Optical Information (Beijing Jiaotong University), Ministry of Education, Beijing, 100044, China.

[2] Institute of Optoelectronics Technology, Beijing Jiaotong University, Beijing, 100044, China.

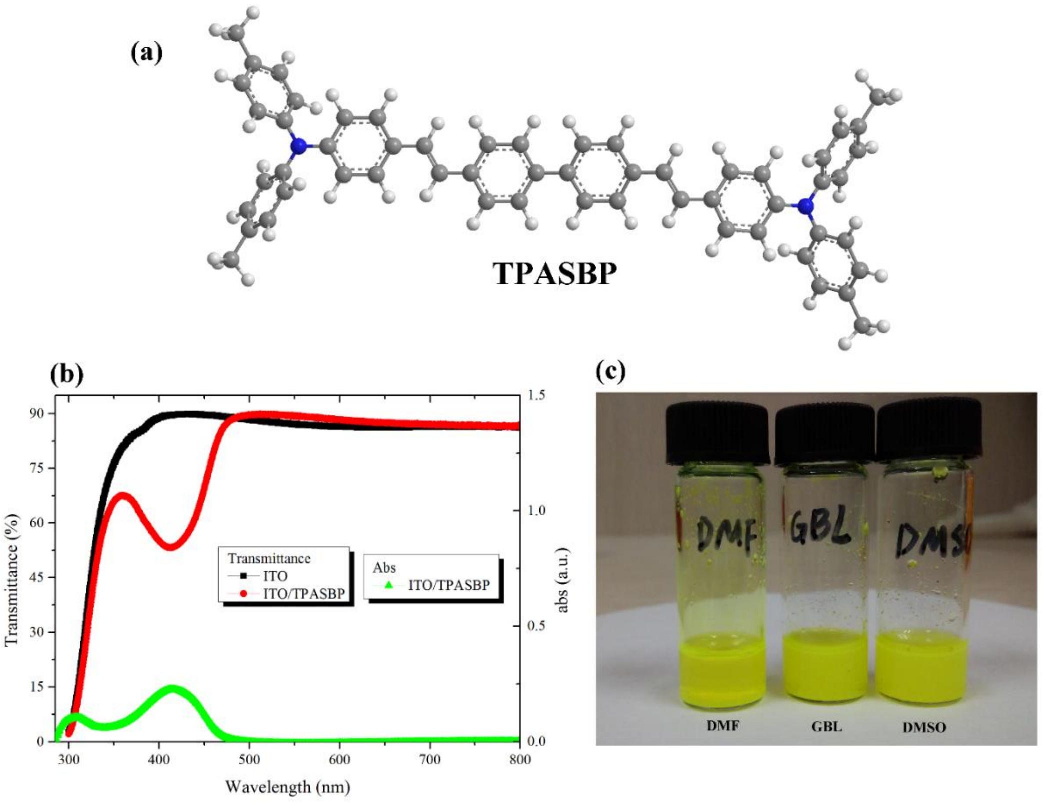

Figure S1. (a) chemical structure of TPASBP, (b) transmittance spectra of TPASBP/ITO grass with ITO/grass as reference and UV-vis absorption spectra of TPASBP on ITO, (c) TPASBP solutions in DMF, GBL, and DMSO, respectively, with the concentration of 5 mg/ml.

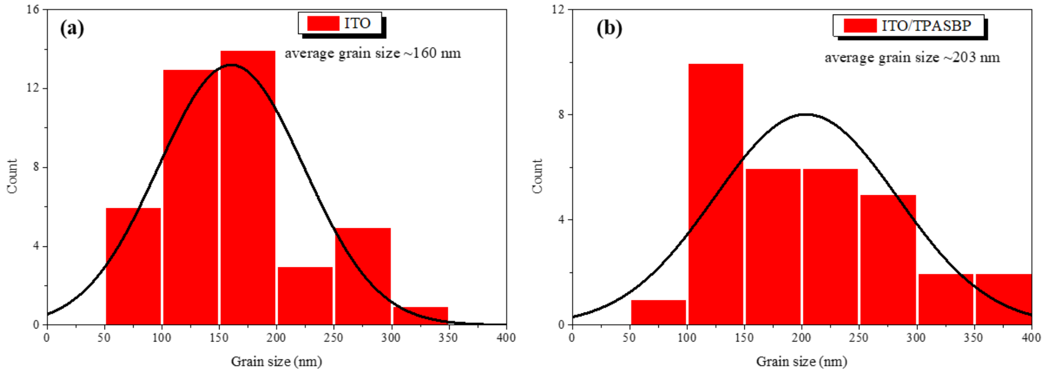

Figure S2. (a) grain size distribution of the perovskite film in Figure 1(c), (b) grain size distribution of the perovskite film in Figure 1(d).

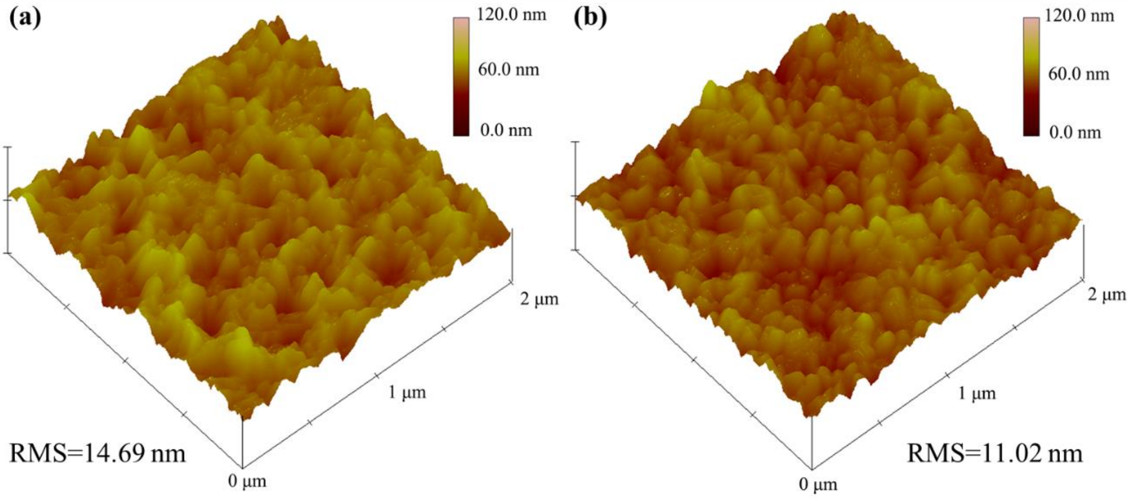

Figure S3. 3D-AFM topography images of the perovskite films deposited on (a) bare and (b) TPASBP-covered ITO substrates.

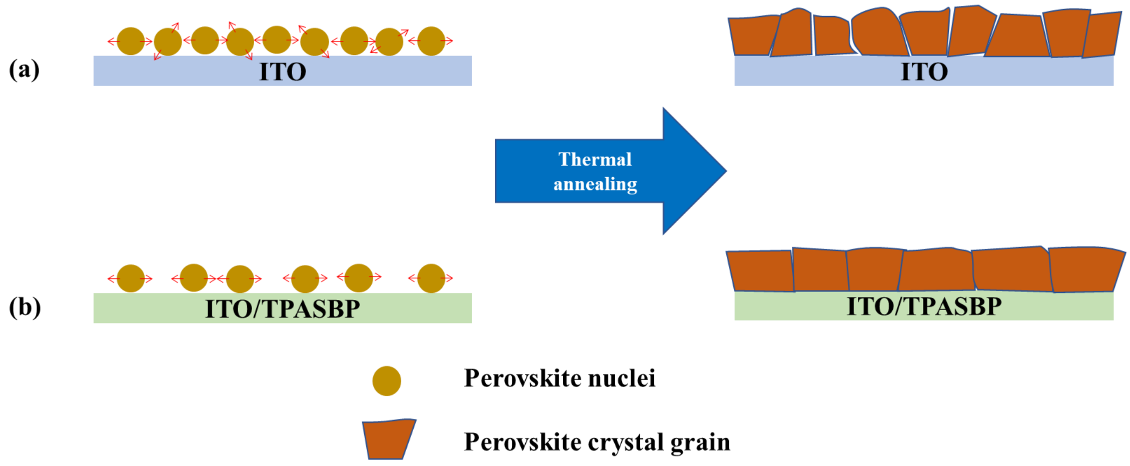

Figure S4. Depiction of the growth process for perovskite films on (a) hydrophilic ITO and (b) hydrophobic TPASBP.

Table S1. Time resolved PL measurements.

Samples	τ_1 (ns)	fraction 1 (%)	τ_2 (ns)	fraction 2 (%)	Averaged time (ns)
ITO/$CH_3NH_3PbI_3$	0.61	8.35	35.15	91.65	32.26

**ITO/TPASBP/$CH_3NH_3PbI_3$**	0.05	15.52	25.88	84.48	21.87

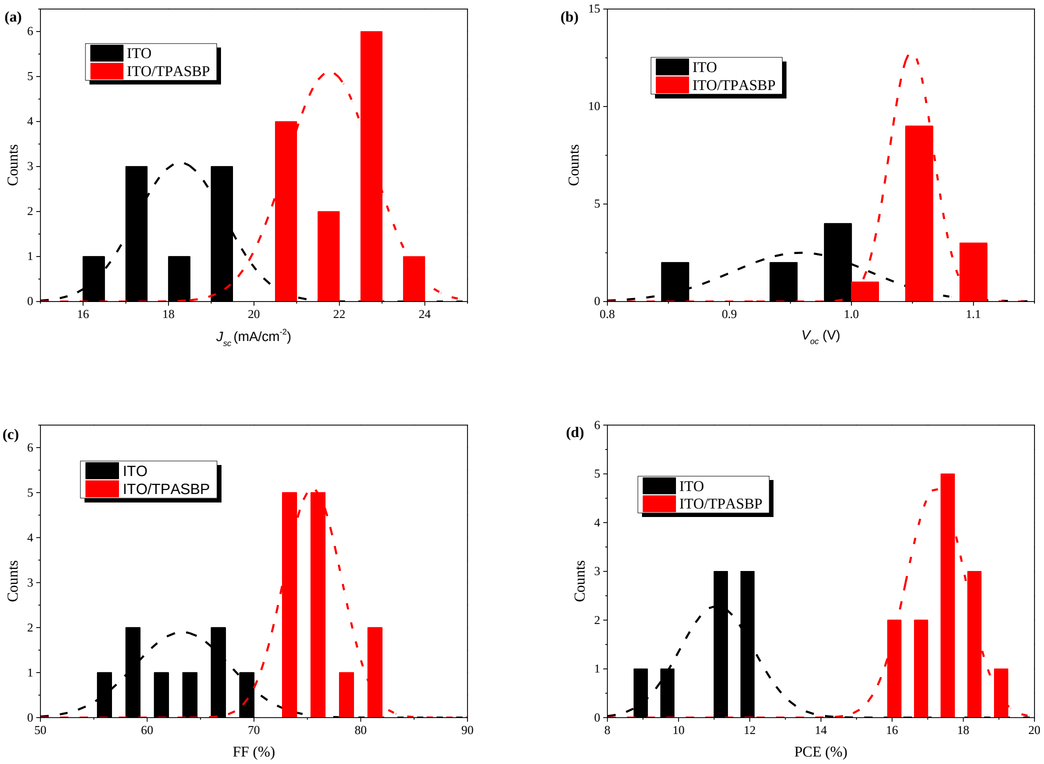

Figure S5. Distributions of (a) Jsc, (b) Voc, (c) FF, and (d) PCE obtained from 8 identical cells for PSCs based on ITO and 13 identical cells for PSCs based on TPASBP.

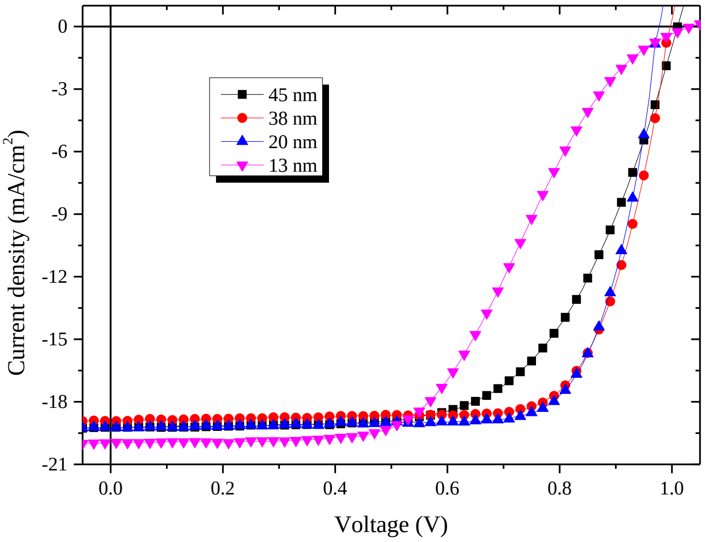

Figure S6. Dependence of main parameters of the device performance on the thickness of TPASBP layer.

Table S2. The photovoltaic parameters of PSCs with different thickness of TPASBP.

Thickness	V_{oc} (V)	J_{sc} (mA/cm^2)	FF (%)	PCE (%)
**45 nm (1500 rpm)**	1.01	19.24	62.18	12.08
**38 nm (2000 rpm)**	1.00	18.94	73.88	13.99
**20 nm (3000 rpm)**	0.98	19.29	75.15	14.21
**13 nm (4000 rpm)**	1.03	19.97	49.70	10.22
